# Foreign Body Aspiration in Northern Ghana: A Review of Pediatric Patients

**DOI:** 10.1155/2017/1478795

**Published:** 2017-10-01

**Authors:** Theophilus Adjeso, Michael Chanalu Damah, James Patrick Murphy, Theophilus Teddy Kojo Anyomih

**Affiliations:** ^1^Department of Eye, Ear, Nose and Throat, School of Medicine and Health Sciences, University for Development Studies, Tamale, Ghana; ^2^Ear, Nose and Throat Unit, Tamale Teaching Hospital, P.O. Box 16, Tamale, Ghana; ^3^Department of Surgery, Tamale Teaching Hospital, P.O. Box 16, Tamale, Ghana

## Abstract

**Background:**

Foreign body (FB) aspiration requires a high index of suspicion for diagnosis and prompt management to avoid morbidity and mortality. This retrospective study was conducted to review pediatric foreign body aspiration at the Ear, Nose and Throat (ENT) Unit of the Tamale Teaching Hospital (TTH).

**Materials and Methods:**

The theater records of children managed for foreign body aspiration from January 2010 to December 2016 at the ENT Unit of TTH were retrieved and data summarized with respect to age, gender, indications for bronchoscopy, nature of foreign body, location of foreign body, and outcome of the bronchoscopy procedure.

**Results:**

A total of 33 children were managed within the five-year study period and comprised 16 (48.5%) males and 17 (51.5%) females. The commonly aspirated FBs were groundnuts (13, 39.4%) and metallic objects (7, 21.1%). The peak incidence occurred in children aged ≤ 3 years. The foreign bodies (FBs) were commonly localized to the right (24.2%) and left (24.2%) main bronchi, respectively. One patient had emergency tracheostomy for failed bronchoscopy.

**Conclusion:**

Groundnuts were the most commonly aspirated foreign body with most of the FBs localized in the bronchi.

## 1. Introduction

Foreign body aspiration occurs mostly in children due to the lack of molar teeth to properly grind food and the tendency to play or talk with food in the mouth; it can, however, also affect adolescents and adults [1, 2]. The gold standard for treatment of foreign body aspiration is rigid bronchoscopy with forceps removal even though flexible bronchoscopy is quite useful in certain conditions [3]. However, in cases of failed rigid bronchoscopy, the surgical options available for retrieving the foreign body include tracheostomy, bronchotomy, and thoracotomy [4–8]. The mortality rate following surgery varies, ranging from 0 to 2.6% [5, 9].

Previous studies in Ghana had shown that fishbone, groundnuts, seeds, plastic materials, and metallic materials were the most commonly aspirated foreign bodies with a majority of them localized to the right main bronchus [4, 10, 11]. The common clinical presentations associated with FB aspiration are respiratory distress, irritating cough, choking, painful swallowing, dysphonia, and stridor. Common radiological findings of aspirated foreign bodies include radiopaque FB, lung consolidation, lung collapse, mediastinal shift, or a normal radiogram [12–14].

In a similar study conducted in India, groundnuts were the most commonly aspirated foreign body with cough and wheezing as well as reduced breath sounds being the most common clinical presentation [15]. The majority of the foreign bodies were found to be localized in the right main bronchus in this study. Despite reported studies of the right main bronchus being the most common site for these foreign bodies, other authors noted the left main bronchus to be the most common site of lodgment of foreign bodies [16]. This is because the differences between the right and left bronchi are less pronounced in children compared with adults [15].

Prior to July 2007, patients with aspirated foreign bodies in the three northern regions (Northern, Upper East, and Upper West) of Ghana were referred to tertiary hospitals in the south, notably the Korle-Bu and Komfo Anokye Teaching Hospitals in Accra and Kumasi, respectively, for treatment due to nonavailability of the requisite expertise and equipment to manage these patients. Since then, such patients have been treated successfully at the Tamale Teaching Hospital (TTH), the third largest tertiary health facility in the country situated in the Northern Region.

To date, there are no published reports on aspirated foreign bodies in children in our local setting. This retrospective study was conducted to review all pediatric patients being managed for aspirated foreign bodies at the ENT Unit, Tamale Teaching Hospital, Tamale, Ghana.

## 2. Method

This descriptive retrospective study of foreign body aspiration in children was conducted in the ENT Unit of the Tamale Teaching Hospital (TTH). TTH is the third largest teaching hospital in Ghana, located in the northern region. It serves as a tertiary referral center for the three northern regions and has a bed capacity of 480 and catchment population of approximately 4.3 million people. The major economic activity in the three regions of the north is agriculture. Crops grown include groundnuts, rice, millet, corn, and shea butter trees.

The theater records of all patients managed with direct laryngoscopy/rigid bronchoscopy under general anesthesia on account of foreign body aspiration from January 2010 to December 2016 in the ENT Unit were retrieved. Retrospective data review was commenced from 2010 because the records from that year represented the most complete data set available. Extracted data included age, sex, nature of the foreign body, location of the foreign body, and outcomes of direct laryngoscopy/bronchoscopy. Approval to conduct this retrospective institutional review was obtained from the Ethical Review Board of the Tamale Teaching Hospital.

All extracted data was entered into Microsoft Excel 2010 and appropriately cleaned for double entry and typographical errors. Cleaned data was then exported into SPSS version 20 (Chicago, IBM, 2010) for descriptive statistical analysis using means, median, standard deviation, and frequencies.

## 3. Results

Over the five-year study period, a total of 33 children were managed for FB aspiration as indicated from the retrieved records. These comprised 16 males (48.5%) and 17 females (51.5%) giving a male-to-female ratio of approximately 1 : 1. The median age of the children was 2 years with age range between 7 months and 10 years. On stratifying age into three age groups (≤3, 4–6, and 7–10 years, resp.), approximately 70% of the foreign body aspirations occurred within the ≤3-year age group followed by the 4–6-year age group (18.2%) (Table 1).

The commonest foreign bodies aspirated were groundnuts (13, 39.4%), metallic objects (7, 21.2%), and seeds (3, 9.1%) (Table 2).

**Figure 1:**
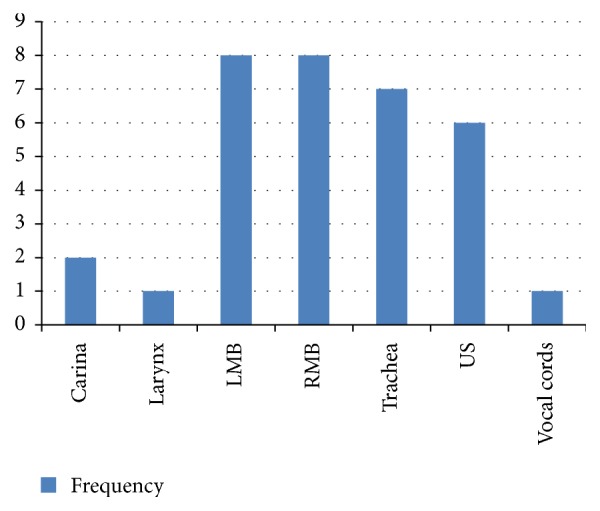
Locations of the aspirated foreign bodies.

The foreign bodies were localized to the right main bronchus in 8 (24.2%) patients, left main bronchus in 8 (24.2%) patients, and trachea in 7 (21.2%) patients. Two (6.1%) each of the foreign bodies were localized to the larynx and carina, respectively, but in 6 (18.2%) patients the location of the foreign bodies was unspecified (US) (Figure 1).

Emergency tracheostomy was done to retrieve a FB in one of the patients on account of failed rigid bronchoscopy. The remaining patients had their FBs removed successfully using Storz laryngoscopes, ventilating bronchoscopes, and telescopic forceps with the image projected via an attached Dyonics camera unto a Sony monitor with little mucosal damage. No mortality was recorded during rigid bronchoscopy.

## 4. Discussion 

From our review, foreign body aspirations were predominately common in children three years of age and less. This finding is similar to other series in the literature [15–20]. The observed male-to-female ratio of approximately 1 : 1 in this study is also similar to that reported by a study in Accra, Ghana [4]. The most commonly aspirated foreign bodies in this review were similar in terms of classification as organic and inorganic foreign bodies compared with findings by other authors [4, 10, 17–20].

The most common foreign bodies encountered included groundnuts, metallic objects, seeds, fishbone, plastic objects, grains, and cartilage. Our findings are consistent with other published series [4, 10, 11, 18, 21]. Groundnuts were the most commonly aspirated foreign body in our study because most parents and guardians of such children were either farming or trading in groundnuts. In addition, groundnuts are a social delicacy within the study area, which are eaten either boiled or roasted. Furthermore, it is not uncommon to find adults handing over one or two of these nuts to children to play with, knowing quite well that they would be unable to chew them properly and successfully.

The majority of the foreign bodies were localized to the two main bronchi, which was found to be consistent with the findings of Girardi et al. [14]. However, the present retrospective review observed a 21.2% localization of FBs in the trachea and 6.1% localization in the larynx compared to the 4.5% trachea localization and 6% larynx localization in the study by Girardi et al. (2004).

Emergency tracheostomy with rigid bronchoscopy was done for one patient who had a failed rigid bronchoscopy due to the inability of the foreign body to pass through the subglottic region [5]. The tracheostomy was maintained with a size 4 plastic uncuffed tracheostomy tube for 24 hours to allow for edema to subside. The patient was discharged on the second postoperative day. Bronchotomy and thoracotomy are the other options available for cases of failed bronchoscopy [4, 6–8]. No mortality was recorded during rigid bronchoscopy.

Our study did not evaluate the clinical presentations and radiological findings of the FB aspirations, which is a limitation; nonetheless, it would serve as a baseline for future prospective studies.

Parents and guardians should be educated about the potential dangers of aspirating foreign bodies, especially in very young children, and the need to create a safe environment for them. A prospective study on FB aspirations is strongly recommended.

## 5. Conclusion

The majority of FB aspirations occurred in children aged three years or less with both sexes affected equally. Groundnuts were the most commonly aspirated foreign bodies with a majority being localized in the bronchi. In majority of the patients, the foreign bodies were retrieved successfully by rigid bronchoscopy.

## Figures and Tables

**Table 1:** Age and sex distribution of patients.

Age group (years)	Male (*N*; %)	Female (*N*; %)	Total (*N*; %)
0–3	12 (75.0)	11 (64.7)	23 (69.7)
4–6	1 (6.3)	5 (29.4)	6 (18.2)
7–10	3 (18.7)	1 (5.9)	4 (12.1)

	**16 (44.5)**	**17 (51.5)**	**33 (100.0)**

**Table 2:** Nature of aspirated foreign bodies.

Foreign body	Number	Percentage (%)
Groundnuts	13	39.4
Fishbone	3	9.1
Cartilage	2	6.1
Maize	2	6.1
Metallic object	2	6.1
Plastic pen cap	2	6.1
Spring coil	2	6.1
Board paperclip	1	3.0
Ebony seed	1	3.0
Gaya seed	1	3.0
Hair clip	1	3.0
Pin	1	3.0
Seed	1	3.0
Stone	1	3.0

**Total**	**33**	**100.0**
